# Reply to ‘Critical appraisal of an article on factitious schizophrenia’

**DOI:** 10.4103/0019-5545.31589

**Published:** 2006

**Authors:** Suresh Kumar, Sandeep Grover, S.K. Mattoo

**Affiliations:** * Assistant Professor, Department of Psychiatry, Post Graduate Institute of Medical Education and Research (PGIMER), Chandigarh; ** Senior Resident, Department of Psychiatry, Post Graduate Institute of Medical Education and Research (PGIMER), Chandigarh; *** Additional Professor, Department of Psychiatry, Post Graduate Institute of Medical Education and Research (PGIMER), Chandigarh

Sir,

We are grateful to Raj and Sidhu^1^ for their ‘Critical appraisal…’ of our case report.^2^

We agree that there is nothing unique about the existence of feigned psychosis as much as there is nothing unique about the opinion questioning its diagnostic validity. We had reviewed the scientific literature both for and against these positions. We think that Raj and Sidhu's conclusion that the phenomenological construct in our report is fraught with errors is erroneous.

To begin with, they comment that age of onset, diagnosis and duration of treatment were not properly delineated. Our report mentioned the current age of 18 years (page 169, para 5), the first psychiatric contact at the age of 16 years when a diagnosis of depression was made and antidepressants instituted (page 170, para 4), followed by contact with our services 2 years earlier when a diagnosis of schizophrenia was made and antipsychotics instituted (page 169, para 6). The diagnosis of factitious schizophrenia was made during the current admission at the age of 18 years, making the duration of illness of factitious schizophrenia as approximately 2 years (page 169, para 6).

Regarding the transient blindness, we agree with the differential diagnosis and the line of investigations suggested by Raj and Sidhu. We considered the possibilities of dissociative blindness, occipital seizure, factitious disorder and malingering for the above symptoms. Further, the description and management of transient blindness in this patient needs to be understood from three other perspectives. First, it was reported to us only during the index admission when the patient was diagnosed as a case of factitious disorder. Second, at the time of manifestation of transient blindness, the patient was managed at the emergency setting of some other hospital. Third, at the index evaluation he was diagnosed as having seizure disorder and treated with sodium valproate. At the time of the index admission, the patient was reviewed by the neurologist. The history of seizure being consistent and the patient already being on antiepileptic drugs obviated the need for EEG. We concluded that the transient blindness was a dissociative symptom—a not so uncommon presentation in factitious disorders.^3^

Raj and Sidhu^1^ have raised queries about the patient's relationship with family, bonding and attachment, stress handling and genetic load. We did mention parental psychological and sexual problems (page 170, paras 3 and 7), mother's dysthymia and antidepressant treatment for the preceding 4 years (page 170, para 3) and his dependency on mother (page 170, para 6). Our statement ‘no problem in social and interpersonal functioning’ must be seen in the context of no gross problems having been reported before the onset of his problems 2 years ago, although we did mention that since the age of 11 he had been preoccupied with parental sexual behaviour/problems and its consequences on the mother, had felt like saving his mother, had angry outbursts against his father, and had been stressed in terms of experiencing anxiety, decreased sleep, academic decline, masturbatory guilt and suicidal behaviour (page 170, paras 5 and 7). His academic decline must be seen in the context of both the emotional turmoil he was passing through and his IQ of 77 (page 170, para 5). The relationship between the diagnosis of an emotionally unstable personality disorder and lack of any gross social and interpersonal problems must be seen in the context of his age of 18, when the personality is considered to be still evolving and a shorter duration of personality disorder may not necessarily lead to too many behavioural and interpersonal problems compared to those seen in patients who are middle aged with long-standing personality disorders.

We accept that we failed to mention the negative history of drug and substance abuse. However, the sexual history was provided in terms of his masturbatory guilt and also his mental status and physical examination findings were mentioned (page 169, para 5).

We diagnosed the subject as having factitious disorder, with predominantly psychological symptoms. It needs to be understood that over the years the nosology of factitious disorders has been better defined and the term Munchausen syndrome is used for only severe forms of factitious disorders. According to the current nosological systems (ICD-10 and DSM-IV), to diagnose a case as factitious disorder, it is not necessary that the subject should have dramatic presentation, pseudologia fantastica and wandering—features listed out by Raj and Sidhu as the pointers against the diagnosis of factitious disorders. It is evident that our patient feigned the symptoms due to psychological distress arising out of his family problems. Once he was diagnosed as a case of schizophrenia, his father's attitude towards him and his mother changed, and this led to his continuation of sick role and knowingly feigning the symptoms. Further, there was an absence of external incentive in the form of economic gain, avoidance of legal responsibility or improving physical well-being as seen in malingering. Also, Raj and Sidhu's contention is erroneous that the patient did not fulfil the criteria for factitious disorder because of the unintentional production of symptoms, motivation to assume a sick role and presence of secondary gain. As described in the case report, the patient admitted in subsequent interviews that he had intentionally continued to feign symptoms, with an attendant unconscious primary gain (relief of mother being protected) of which he became aware only later, during the psychotherapy sessions.

Raj and Sidhu's contention that feigned schizophrenia is only a prodrome of psychosis in extremely deviant premorbid personalities or the borderline personality disorder is an issue that is still open to debate.^3^ Lastly, as described in the case report, the fact that the patient has been following up with us and maintaining well for the past 2 years without any antipsychotic drugs provides further credence to the diagnosis of factitious disorder and rules out the possibility of any prodrome of psychosis.
